# Influence of hydrological conditions on the *Escherichia coli *population structure in the water of a creek on a rural watershed

**DOI:** 10.1186/1471-2180-10-222

**Published:** 2010-08-19

**Authors:** Mehdy Ratajczak, Emilie Laroche, Thierry Berthe, Olivier Clermont, Barbara Pawlak, Erick Denamur, Fabienne Petit

**Affiliations:** 1 Laboratoire M2C, Université de Rouen, CNRS UMR 6143, FED SCALE 4116, 76821 Mont Saint Aignan, France; 2 Laboratoire Ecologie et évolution des microorganismes, Université Paris 7 Denis Diderot and INSERM U722, 75018 Paris, France

## Abstract

**Background:**

*Escherichia coli *is a commensal bacterium of the gastro-intestinal tract of human and vertebrate animals, although the aquatic environment could be a secondary habitat. The aim of this study was to investigate the effect of hydrological conditions on the structure of the *E. coli *population in the water of a creek on a small rural watershed in France composed of pasture and with human occupation.

**Results:**

It became apparent, after studying the distribution in the four main *E. coli *phylo-groups (A, B1, B2, D), the presence of the *hly *(hemolysin) gene and the antibiotic resistance pattern, that the *E. coli *population structure was modified not only by the hydrological conditions (dry versus wet periods, rainfall events), but also by how the watershed was used (presence or absence of cattle). Isolates of the B1 phylo-group devoid of *hly *and sensitive to antibiotics were particularly abundant during the dry period. During the wet period and the rainfall events, contamination from human sources was predominantly characterized by strains of the A phylo-group, whereas contamination by cattle mainly involved B1 phylo-group strains resistant to antibiotics and exhibiting *hly*. As *E. coli *B1 was the main phylo-group isolated in water, the diversity of 112 *E. coli *B1 isolates was further investigated by studying *uidA *alleles (beta-D-glucuronidase), the presence of *hly*, the O-type, and antibiotic resistance. Among the forty epidemiolgical types (ETs) identified, five *E. coli *B1 ETs were more abundant in slightly contaminated water.

**Conclusions:**

The structure of an *E. coli *population in water is not stable, but depends on the hydrological conditions and on current use of the land on the watershed. In our study it was the ratio of A to B1 phylo-groups that changed. However, a set of B1 phylo-group isolates seems to be persistent in water, strengthening the hypothesis that they may correspond to specifically adapted strains.

## Background

Ensuring the high microbiological quality of environmental water used as a source of recreational or drinking water is an important worldwide problem [1]. Poor microbiological quality of water results from contamination by microorganisms of human or animal fecal origin, and leads to the risk of gastro-enteritis in humans. Such contamination is caused by fecal bacteria from (i) point source pollution, e.g., treated effluents from wastewater treatments plants (WWTPs) which primarily treat wastewater of human origin, or (ii) nonpoint source pollution consisting of inputs of microorganisms of mainly animal origin, via run-off or leaching from pasture or manured soils [2-4]. The World Health Organization and, more recently, European guidelines (2006/7/EC), use *Escherichia coli *as the bacterial indicator species for fecal contamination of water. Epidemiological studies have been used to determine threshold values for concentrations of *E. coli *in water above which there is a risk of gastro-enteritis [5-7].

*E. coli *is a commensal bacterium of the gastro-intestinal tract of humans and vertebrate animals [8,9]. To survive in an aqueous environment it must resist environmental stressors (oligotrophy, UV, temperature, salinity) [10-12] and avoid predation by protozoa [13]. Some authors have suggested that some of these *E. coli *strains might then persist by becoming naturalized in fresh water and soil [14-16]. The aquatic environment can thus be considered a secondary habitat, where some authors have even shown the possible growth of *E. coli *[17,18]. The diversity of *E. coli *populations in their secondary habitats has been studied by analyzing the sequences of the gene *uidA *[19,20], palindromic repetitive sequences [21,22], ribotypes [23], and profiles of antibiotic resistance [24,25]. Using these methods, the dynamics of *E. coli *populations have been investigated and, in some cases, it has been possible to discriminate between the human or animal origin of the contamination.

The structure of an *E. coli *population is characterized by four main phylo-groups (A, B1, B2, and D) [26-28]. Strains of the phylo-groups A and B1 are mainly found as commensals in humans and vertebrate animals, with the A phylo-group strains being predominant in humans and the B1 strains in animals [29]. Extraintestinal infections are mainly caused by the strains of the phylo-groups B2 and D [30]. Although strains of the B2 and D phylo-groups are typically less abundant as commensals, the distribution of the four phylo-groups can vary according to diet or climate [9,31-33]. It also has been suggested that some strains could be host-specific, such as B1 strains exhibiting the *hly *(hemolysin) gene, found only in animals, and B2 O81 O-type strains, found only in humans [34,35].

**Figure 1:**
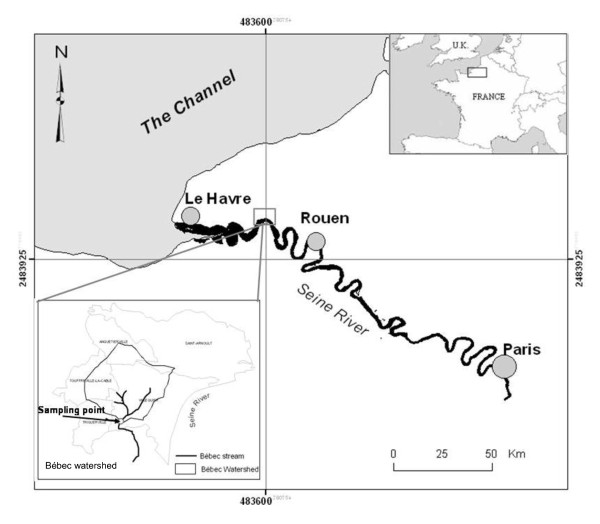
**Location of study site and sample collection point**.

The objective of this study was to investigate the effects of various hydrological conditions on the structure of the *E. coli *population collected from stream water in a small rural watershed in northern France (Figure 1). Land use in the watershed is almost entirely agricultural with a low population density. Results show that an increase of fecal contamination was accompanied by a change in the distribution of phylo-groups in the *E. coli *population, represented by a change in the ratio of A to B1 phylo-groups. *E. coli *B1 isolates were the dominant phylo-group isolated in the water. Among *E. coli *B1 isolates, some epidemiological types (ETs) seem to be specific to water that is only slightly contaminated.

## Results and discussion

### *E. coli *population structure in creek water in relation to hydrological conditions and watershed land use

*E. coli *were enumerated and the population structure analyzed by phylo-grouping in three sets of samples collected under different hydrological and agricultural land-use conditions (Table 1). In this study, the *E. coli *population structure in creek water is analyzed from a single sample integrating all the daily samples. The origin (animal or human) of specific strains was investigated, in addition to the phylo-grouping, by *hly *gene detection in the *E. coli *B1 isolates and O81 typing of *E. coli *B2 isolates, as well as by studying the antibiotic resistance pattern. Statistical analyses (Chi^2 ^test) were performed in order to compare hydrological conditions (dry versus wet periods, rainfall events).

**Table 1:** *E. coli *enumeration in creek water according to land use in the watershed, and hydrological parameters.

		Hydrological conditions				**Use of the watershed**^**a**^	*E. coli*
	**Sampling date** **(day/mo/yr)**	**Rainfall (mm)**		**Turbidity** **(NTU**^**b**^**)**	**SSC**^**c**^ **(mg.L**^**-1**^**)**	**Head of cattle**	**CFU/100 ml**
		**Within 5 days of sampling**	**On day of sampling**				

Wet period	21 Feb 2007	27.8	2.0	15.0	23.0	0	(1.0 ± 0.1) 10^2^
Dry period	3 May 2007	3.8	0.0	3.1	11.4	172	(6.2 ± 0.6) 10^2^
Rainfall event during dry period	11 July 2007	8.9	50.0	33.0	74.4	172	(4.0 ± 0.7) 10^4^

^a ^49 septic tanks (147 eq. inhabitants) were located between 500 to 600 m from the creek. One malfunctioning septic tank (4 eq. inhabitants) was located 400 m from the sampling point.

^b ^Nephelometric turbidity unit

^c ^Suspended Sediment Concentration

During the dry period (May 2007), when cattle were being grazed, but when there was no runoff or leaching, the water was slightly contaminated by *E. coli *(6.2 10^2 ^CFU/100 ml) (Table 1). The structure of the *E. coli *population was significantly different from the structures analyzed from the other sample collection periods (χ^2 ^test P < 0.001), with a majority of *E. coli *B1 isolates (87%) (Table 2). This structure argues for contamination by *E. coli *B1 isolates that are better adapted to the aquatic environment [15], rather than for residual bovine fecal contamination, as the isolates were devoid of the *hly *gene and sensitive to all antibiotics [35,36].

**Table 2:** Structure and antibiotic resistance of the *E. coli *population in the stream during different hydrological conditions (χ^2 ^test *P *< 0.001 ***α = 0.01).

	*E. coli *phylo-group distribution													
	**A**			**B1**				**B2**				**D**		

**Hydrologic conditions**	**%** **(n)**	**Numbers of antibiotic-resistant**^**a**^	**Antibiotic** **resistance**^**b **^**(n)**	**%** **(n)**	***hly***^***c***^	**Numbers of antibiotic-resistant**^**a**^	**Antibiotic** **resistance**^**b **^**(n)**	**%** **(n)**	**O81**^**d**^	**Numbers of antibiotic-resistant**^**a**^	**Antibiotic** **resistance**^**b **^**(n)**	**%** **(n)**	**Numbers of antibiotic-resistant**^**a**^	**Antibiotic** **resistance**^**b**^ **(n)**

**Wet period**	47% (21)***	0	nd	39% (17)***	0	0	nd	7% (3)	0	0	nd	7% (3)	0	nd

**Dry period**	7% (3)***	0	nd	87% (39)***	0	0	nd	2% (1)	0	0	nd	4% (2)	0	nd

**Rain event during dry period**	32% (11)	7	CHL(3) TET(3) STR(1)	44% (15)	2	10	CHL (5) TET(3) CHL/TET(2)	0% (0)	nd	nd	nd	23% (8)	2	CHL/TET(1) CHL(1)

n: numbers of isolates

^a ^*E. coli *isolates resistant to one or more antibiotics

^b ^CHL: chloramphenicol; TET: tetracyclin; STR: streptomycin

nd: not detected

^c ^*hly *gene detection by PCR method

^d ^Serotype O81 detection by PCR method

It was during the wet period (February 2007), when there was no grazing, but when there was a malfunctioning septic system (4 equivalent inhabitants), that the lowest value of *E. coli *(1.0 10^2 ^CFU/100 ml) was measured in the water. The *E. coli *population was characterized by a high proportion of phylo-group A isolates (47%) (χ^2 ^test P < 0.001), followed by *E. coli *B1 isolates without the *hly *gene (Table 2). None of the *E. coli *was resistant to the seven antibiotics tested (Table 2). This *E. coli *population is probably due to an input of solely human origin, as the structure corresponds to that already described for human commensal *E. coli *in France [31,32].

The rainfall event that occurred during the dry period (July 2007) resulted in runoff from the pastures and leaching of soils. The density of the *E. coli *in the stream water (4.0 10^4 ^CFU/100 ml) was two orders of magnitude higher than that measured for the two other periods (Table 1). During this rain event, an input of *E. coli *from cattle contamination (172 head of cattle) was added to that from human contamination (147 eq. inhabitants, 49 septic tanks, and the malfunctioning septic tank). The structure of the *E. coli *population was characterized by two main phylo-groups, B1 (44%) and A (32%). Some *E. coli *B1 isolates with the *hly *gene, presumably of animal origin were detected (2/15) [35]. More than 60% of these isolates were resistant to at least one of the three antibiotics used in veterinary medicine (chloramphenicol, tetracycline, and streptomycin) [37] (Table 2), suggesting an animal origin.

Thus, it appears that both hydrological conditions and current land use in the watershed might affect the structure of the *E. coli *A and B1 populations in the stream. In contrast, the hydrological and land-use conditions did not exert a significant influence on the phylo-groups B2 and D, which were the least abundant phylo-groups recovered from the water (between 0 and 23%). No human-specific B2 O81 O-type strain was isolated during any sampling conditions, which is consistent with the low frequency of these strains in the *E. coli *population [34].

### Changes in *E. coli *population structure during a rain event

**Figure 2:**
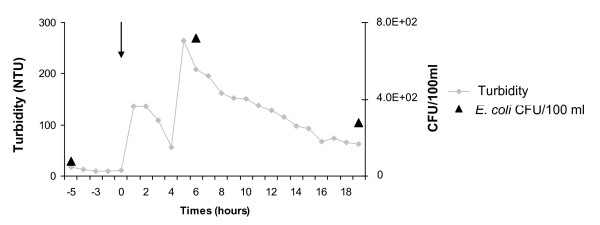
**Influence of a rain event during a wet period on *E. coli *density**. The arrow indicates the beginning of 14 mm rain event.

In order to better understand the effect of a rain event on the structure of an *E. coli *population, we selected three out of the twenty-four hourly samples. Our selection represented three key moments (5 hours before, 6 hours after, and 19 hours after the rain event) showing how the turbidity and *E. coli *density evolved. It would not have been possible to observe this evolution using just a sample that integrated all the daily samples. The rain event consisted of 14 mm of rain that fell during a wet period, during which there were 42 cattle being grazed in the watershed (March 2008) (Figure 2). Five hours before rainfall began, the level of *E. coli *contamination was low (7.6 10^1 ^CFU/100 ml), and the small number of isolates did not permit analysis of the structure of the *E. coli *population (Table 3). During the rain event, the turbidity increased, as did the number of *E. coli*, consistent with previous work demonstrating a correlation between bacteria and particles [38]. Six hours after the rainfall event the *E. coli *density reached a value of 7.2 10^2 ^CFU/100 ml, at which point the structure of the *E. coli *population was characterized by a majority of *E. coli *phylo-group A (56%), with 63% being resistant to at least one antibiotic (amoxicillin, chloramphenicol, tetracycline, and streptomycin), suggesting fecal contamination of human origin resulting from leaching of soils and from surface runoff (Table 3). This structure was significantly different from that observed in the less contaminated water analyzed 19 hours after the rainfall event (χ^2 ^test P < 0.001). At that time the *E. coli *density had decreased to 2.8 10^2 ^CFU/100 ml (Figure 2), and *E. coli *B1 isolates (74%) were the predominant *E. coli *phylo-group. These isolates are mainly *hly *positive (72%) with 31% resistant to at least one antibiotic (amoxicillin, tetracycline, and chloramphenicol), suggesting that there had been an input on the soils of *E. coli *of bovine origin that was then introduced into the water through run-off and/or leaching.

**Table 3:** Structure and antibiotic resistance of the *E. coli *population in the stream in response to a rain event (χ^2 ^test *P *< 0.001 ***α = 0.01).

	*E. coli *p-group distribution													
	**A**			**B1**				**B2**				**D**		

**Timing (h)**^**a**^	**%** **(n)**	**Numbers of antibiotic-resistant**^**b**^	**Antibiotic** **Resistance**^**c**^ **(n)**	**%** **(n)**	***hly***^***d***^	**Numbers of antibiotic-resistant**^**b**^	**Antibiotic** **Resistance**^**c**^ **(n)**	**%** **(n)**	**O81**^**e**^	**Numbers of antibiotic-resistant**^**b**^	**Antibiotic resistance**^**c **^**(n)**	**%** **(n)**	**Numbers of antibiotic-resistant**^**b**^	**Antibiotic** **resistance**^**c**^ **(n)**

**-5 h**	25% (3)	2	AMX/CHL(1) CHL(1).	50% (6)	0	4	CHL(4)	8% (1)	0	0	nd	17% (2)	0	nd

**+6 h**	56% (22)***	14	AMX/TIC/CHL(5) AMX/TIC/CHL/SXT/STR(1) AMX/TIC/SXT/STR(1) CHL(6) CHL/TET(1)	15% (6)***	1	3	CHL(3)	8% (3)	0	2	CHL(2).	21% (8)	4	AMX/TIC/SXT/STR(1) CHL(3)

**+19 h**	15% (6)***	3	AMX/CHL(1) AMX/TIC/CHL(1) AMX/TIC(1)	74% (29)***	21	9	TET(1) CHL(7) AMX/CHL/TET(1)	5% (2)	0	2	CHL(2).	5% (2)	1	CHL(1)

nd: not detected

n: numbers of isolates

^a ^Timing in relation to rainfall

^b ^*E. coli *isolates resistant to one or more antibiotics

^c ^AMX: amoxicillin; TIC: ticarcillin CHL: chloramphenicol; TET: tetracyclin; STR: streptomycin; SXT:trimethoprim + sulfamethoxazole

^d ^*hly *gene detection by PCR method

^e ^Serotype O81 detection by PCR method

We cannot exclude an input from wild animals (mainly birds and rabbits), although wild *E. coli *strains are usually not resistant to antibiotics [39]. These results indicate that during the rain event, an increase in microbial contamination was accompanied by a modification of the structure of the *E. coli *population, resulting in a high ratio of phylo-groups A/B1. In contrast, in the water collected 19 h after the rain event, and only slightly contaminated by *E. coli*, the majority of *E. coli *isolates belonged to the B1 phylo-group.

### Diversity of *E. coli *B1 strains isolated from the creek water

As *E. coli *B1 was the dominant phylo-group isolated in water from the Bébec, accounting for between 15% to 87% of the *E. coli *population (Tables 2 and 3), we investigated further the diversity of *E. coli *B1 isolates by (i) sequencing the *uidA *gene (beta-D-glucuronidase, 600 pb) and comparing the sequences obtained with those in the MLST Pasteur database in order to find the *uidA *allele, (ii) detecting the presence of *hly *and determining molecularly the O-type, (iii) studying the antibiotic resistance profile.

**Figure 3:**
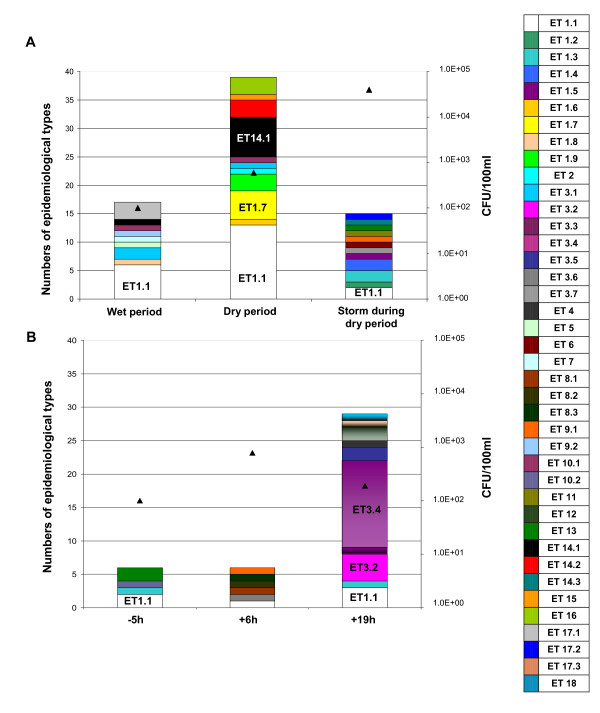
**Distribution of *E. coli *B1 epidemiological types in relation to hydrological conditions (A) and before and after a rain event during a wet period (B)**.

A total of 40 epidemiological types (ETs) were identified among the 112 *E. coli *B1 isolated from the water (Table 4) and the proportion of each ETs differed for each sampling event (Figure 3A and 3B).

**Table 4:** Epidemiological types of *E. coli *B1 strains recovered from creek water.

Epidemiological types	*uidA *allele	*hly*	**Antibiotic**^**a**^			**O-type**^**b**^	Numbers of isolates
			AMX	CHL	TET		
**ET 1.1**	*uidA2*	0	0	0	0	NT	**27**
**ET 1.2**	*uidA2*	**1**	0	0	0	NT	**1**
**ET 1.3**	*uidA2*	0	0	**1**	0	NT	**4**
**ET 1.4**	*uidA2*	0	0	0	**1**	NT	**2**
**ET 1.5**	*uidA2*	0	0	**1**	**1**	NT	**1**
**ET 1.6**	*uidA2*	0	0	0	0	**O8**	**1**
**ET 1.7**	*uidA2*	0	0	0	0	**O15**	**5**
**ET 1.8**	*uidA2*	0	0	0	0	**O26**	**1**
**ET 1.9**	*uidA2*	0	0	0	0	**O40**	**3**
**ET 2**	*uidA4*	0	0	0	0	NT	**1**
**ET 3.1**	*uidA5*	0	0	0	0	NT	**3**
**ET 3.2**	*uidA5*	**1**	0	0	0	NT	**4**
**ET 3.3**	*uidA5*	**1**	0	**1**	0	NT	**1**
**ET 3.4**	*uidA5*	**1**	0	0	0	**O7**	**13**
**ET 3.5**	*uidA5*	**1**	0	**1**	0	**O7**	**2**
**ET 3.6**	*uidA5*	0	0	**1**	0	**O7**	**1**
**ET 3.7**	*uidA5*	**1**	0	0	0	**O88**	**1**
**ET 4**	*uidA11*	0	0	**1**	0	NT	**1**
**ET 5**	*uidA20*	0	0	0	0	NT	**1**
**ET 6**	*uidA21*	0	0	**1**	0	NT	**1**
**ET 7**	*uidA22*	0	0	0	0	**O15**	**1**
**ET 8.1**	*uidA30*	0	0	0	0	**O7**	**1**
**ET 8.2**	*uidA30*	0	0	**1**	0	**O7**	**1**
**ET 8.3**	*uidA30*	**1**	0	0	0	NT	**1**
**ET 9.1**	*uidA50*	0	0	**1**	0	NT	**2**
**ET 9.2**	*uidA50*	0	0	0	0	**O15**	**1**
**ET 10.1**	*uidA55*	0	0	0	0	NT	**2**
**ET 10.2**	*uidA55*	0	0	**1**	0	NT	**1**
**ET 11**	*uidA57*	0	0	0	0	**O8**	**1**
**ET 12**	*uidA65*	0	0	**1**	0	NT	**4**
**ET 13**	*uidA66*	0	0	**1**	0	**O26**	**1**
**ET 14.1**	*uidA90*	0	0	0	0	**O150**	**8**
**ET 14.2**	*uidA90*	0	0	0	0	**O15**	**3**
**ET 14.3**	*uidA90*	0	0	0	**1**	**O26**	**1**
**ET 15**	*uidA103*	0	0	0	0	NT	**1**
**ET 16**	*uidA110*	0	0	0	0	NT	**3**
**ET 17.1**	*uidA111*	0	0	0	0	NT	**3**
**ET 17.2**	*uidA111*	0	0	**1**	**1**	NT	**1**
**ET 17.3**	*uidA111*	0	**1**	**1**	**1**	NT	**1**
**ET 18**	New allele	**1**	0	0	**1**	**O7**	**1**

^a^AMX: amoxicillin; CHL: chloramphenicol; TET: tetracyclin; all of epidemiological types of *E. coli *B1 strains were non-resistant to TIC: ticarcillin; SXT: trimethoprim + sulfamethoxazole; STR: streptomycin; and CIP: ciprofloxacin.

^b^NT: not O7, O8, O15, O26, O40, O45b, O78, O81, O88, O103, O104, O111, O128, or O150.

The binary coding stands for presence (1) or absence (0) of *hly *gene amplification, and resistance (1) or non-resistance (0) to antibiotics.

In the most contaminated water (4.0 ± 0.7 10^4 ^CFU/100 ml), the diversity of *E. coli *B1 strains (i.e., number of ETs/total number of B1 isolates for the sampling campaign) was higher (12/15) than in less contaminated water (9/17 in water containing 1.0 ± 0.1 10^2 ^CFU/100 ml; 12/39 in water containing 6.2 ± 0.6 10^2 ^CFU/100 ml) (Figure 3A). At the peak of the turbidity, *E. coli *density reached a value of 7.2 10^2 ^CFU/100 ml, the diversity of *E. coli *B1 strains was higher (6/6) than the diversity observed when turbidity and *E. coli *density decreased (10/29) (Figure 3B).

Among the 40 ETs, strains of the group ET1.1 were present in all samples, regardless of the hydrological condition or the current land use in the watershed. However, they made up a greater proportion of the strains under non-storm conditions: during the dry period (no contribution of fecal bacteria from the watershed), 13 ET1.1/39 *E. coli *B1 were present, and during the wet period (a low contribution of human-derived fecal material, but none from livestock) 6 ET1.1/17 *E. coli *B1 were present (Figure 3A). In contrast, other ETs were present only under certain hydrological conditions and/or land-use conditions. ET1.7 and ET14.1 were present only during the dry period. ET3.4 was present after the rain event only when the turbidity decreased after the peak had been reached (Figure 3B). These results indicate that specific *E. coli *B1 ETs are more abundant in water that is only slightly contaminated, suggesting better survival of these ETs. These results strengthen the hypothesis of Walk *et al*., [15], that some strains of *E. coli *B1 phylo-group are persistent in water and might correspond to strains with an adaptive advantage in water. However, it must be pointed out that in this work, the *E. coli *A_0 _isolates (50/213), without any amplification of the genes *chuA*, *yjaA *and the fragment TSPE4.C2, could correspond to the new clades of *Escherichia *recently described which appear to be environmentally adapted [40].

## Conclusions

In environmental water, the occurrence of *E. coli*, a bacterial indicator of fecal contamination, is related to both the use of the watershed by livestock and humans combined and the hydrological conditions [2,3,41]. In this study, focused on a small rural watershed composed of pasture and human occupation, we showed that both the number and the structure of the population of *E. coli *were modified by hydrological conditions and use of the watershed. In this watershed, following rainfall, an increase of fecal contamination was accompanied by a modification of the distribution of phylo-groups in the *E. coli *population, represented by change in the ratio of A to B1 phylo-groups. *E. coli *B1 strains were the dominant phylo-group isolated in the water. Among *E. coli *B1 isolates, some ETs seem to be specific to water that is only slightly contaminated, suggesting different survival abilities among *E. coli *B1 strains.

The results from this study do not question the choice of *E. coli *as a bacterial indicator of microbial quality of water DCE 2006/7/CE (Excellent quality CFU/100 ml ≤500). They rather indicate that the structure of an *E. coli *population in water is not stable, but depends on the hydrological conditions, on current use of the watershed land, and on both the origin and intensity of the contamination by fecal bacteria.

## Methods

### Study site

The study was carried out in the experimental watershed "Le Bébec" (Haute Normandie, France) (Figure 1). The Bébec stream drains a small watershed of about 10 km^2^, of which 95% is classified as agricultural land. The elevation of the plateau on which Le Bébec is located averages about 100 m. The soils on the plateau consist of silts approximately 10 m thick, and are highly susceptible to crusting, compaction, and erosion, particularly during the autumn and winter. This watershed is located in a temperate zone with an oceanic climate. Annual precipitation during the period of the study was 1012 mm, and the daily average temperature was 10.9°C. Flow in the Bébec varied from 3 l.s^-1 ^in summer dry periods to 15 l.s^-1 ^in winter, and reached up to 500 l.s^-1 ^in response to major winter storms. Water from the creek recharges the underlying chalk aquifer through a swallow hole. The karstified chalk aquifer has been widely studied [38]. When the flow rate in the stream exceeds the infiltration capacity of the swallow hole, the creek water overflows its banks and floods the valley. Land use in the area consists of approximately 55% cropland, 30% pasture (42 beef cattle, 130 dairy cattle), and 10% forest, with the remaining 5% divided among several other uses. The 213 households in the watershed (639 equivalent inhabitants) rely on on-site septic systems. Among them, 49 septic tanks (147 equivalent inhabitants) were located on a 500 to 600 m stretch of the stream. Untreated sewage of human origin (4 equivalent inhabitants) resulting from a dysfunctional septic system was located 400 m from the sampling location corresponding to a input of *E. coli *which varies from 6.5 10^1 ^CFUs per 100 ml^-1 ^in a wet period to 3.6 10^4 ^CFUs per 100 ml^-1 ^after a rainfall event. The land-use data were provided by the "Groupement d'Intéret Public Seine Aval", and data on beef and dairy cattle were provided by the "Direction Départementale de l'Agriculture et de la Forêt (DDAF)".

### Materials and sampling method

Samples were collected with autosamplers (ISCO 6700 s, Roucaire, Courtaboeuf, France) from the stream, near the swallow hole, during a wet period in February 2007 (high flow) and during a dry period in May 2007 (low flow), after a storm during a dry period in July 2007 (Table 1), and after a storm during a wet period in March 2008, with samples taken 5 h before the storm, 6 h after the storm, and 19 h after the storm (Figure 2). The site was equipped with dataprobes (YSI 6820) to measure turbidity. Suspended sediment concentration was measured by filtration through pre-weighed Millipore filters (0.45 μm). Water (1 L) was collected by autosamplers every hour for 24 h, 250 ml of each flask were mixed until subsequent microbial analysis, except for the sampling campaign in March 2008. All samples were kept at 4°C until the microbiological analyses were carried out, which occurred within 8 h.

### Enumeration of culturable *E. coli*

*E. coli *were enumerated using membrane filtration methods (0.45 μm HA047 Millipore, Bedford, MA, USA). *E. coli *were isolated from the water samples with a selective chromogenic media specific for *E. coli*, with the addition of a selective supplement for water samples (RAPID'E.coli 2 Medium and Supplement; Biorad, USA), and incubated for 24 h at 44°C. The threshold value for the enumeration of *E. coli *in water was 5 CFUs per 100 ml^-1^.

### *E. coli *isolates

Two hundred and thirteen isolates of *E. coli *were isolated from the creek water. The isolates were taken from the membrane of RAPID'E.coli 2 medium and isolated on RAPID'E.coli 2 medium for 24 h at 37°C. Each clone of *E. coli *was stored on a cryo-bead system (AES laboratory, France) at -80°C. Four sets of isolates were obtained from the stream under different hydrological conditions: 44 isolates during dry season conditions (February 2007); 45 isolates during wet season conditions (May 2007); 34 isolates after a storm during the dry period (July 2007); and 90 isolates from the storm during the wet period (March 2008).

### Determination of the *E. coli *phylo-groups, O type, and presence of the *hly *gene

The phylogenetic group to which the *E. coli *isolates belonged was determined by the PCR-based method, as described previously by Clermont *et al. *[42]. A total of 112 isolates of *E. coli *B1 were tested for the virulence factor *hly *by the PCR amplification method as described by Escobar-Páramo *et al. *[34] (hly.1: 5'-AGG-TTC-TTG-GGC-ATG-TAT-CCT-3'; hly.2: 5'-TTG-CTT-TGC-AGA-CTG-CAG-GTG-T-3'). All *E. coli *B2 were tested for the O81 type [10], and all *E. coli *B1 strains were tested for O7, O8, O15, O26, O40, O45b, O78, O81, O88, O103, O104, O111, O128 and O150 types by using the PCR-based method described by Clermont *et al. *[43] with the primers shown in [Additional file 1]. These O types have been previously shown to be present in B1 group strains (Clermont and Denamur, personal data).

### Antibiotic resistance testing

Antibiotic resistance was determined by the agar diffusion method using seven antibiotic disks (BioMérieux, France): amoxicillin (AMX), ticarcillin (TIC), chloramphenicol (CHL), tetracycline (TET), trimethoprim + sulfamethoxazole (SXT), ciprofloxacin (CIP), and streptomycin (STR). Among them CHL, TET, STR are used in veterinary medicine. After 24 h of incubation at 37°C, the bacteria were classified as sensitive, intermediate, or resistant according to French national guidelines [44]. The *E. coli *CIP 7624 (ATCC 25922) was taken as the quality control strain. The data were regrouped as resistant or non-resistant, the latter corresponding to sensitive and intermediate phenotypes.

### Allele number attribution of *uidA *gene of *E. coli *B1

Partial *uidA *sequences (600 pb) of 112 *E. coli *B1 isolates from the stream (17, dry season; 39, wet season; 15, storm during dry period; 41, storm during wet period [6, 6, and 19 5 h before the storm, 6 h after the storm, and 19 h after the storm, respectively]) were sequenced after PCR amplification (uidAR: 5'-CCA-TCA-GCA-CGT-TAT-CGA-ATC-CTT-3'; uidAF:5' CAT-TAC-GGC-AAA-GTG-TGG-GTC-AAT-3'). Thirty-five μl of PCR product, containing an estimated 100 ng/μl of DNA, were sequenced in both forward and reverse directions at Cogenics (Meylan, France). A consensus sequence was determined by aligning the forward sequence with the reverse complement of the reverse sequence. Alleles of *uidA *were determined by comparison of the *uidA *sequences found in the MLST database Pasteur http://www.pasteur.fr/cgi-bin/genopole/PF8/mlstdbnet.pl?file=Eco_profiles.xml.

### Statistical analyses

The frequencies of various phylo-groups in the water samples were compared using the chi-square test. Tests were carried out using the XLSTATS version 6.0 (Addinsoft).

## Abbreviations

AMX: amoxicillin; CFUs: colony-forming units; CHL: chloramphenicol; CIP: ciprofloxacin; ET: epidemiological type; MLST: multi locus sequence typing; PCR: polymerase chain reaction; TET: tetracycline; TIC: ticarcillin; STR: streptomycin; SXT: trimethoprim + sulfamethoxazole.

## Authors' contributions

The work presented here was carried out in collaboration with all authors. MR, TB and FP defined the research theme. MR, TB and FP defined sampling strategy and designed methods and experiments. EL and BP defined sampling strategies during the rain event. MR carried out the laboratory experiments, and EL carried out antibiotic resistance analysis. MR and FP analyzed the data, interpreted the results and wrote the paper. OC and ED co-designed experiments, discussed analyses, interpretation and presentation. All authors have contributed to, seen and approved the final manuscript.

## Supplementary Material

Additional file 1 **List of primers used in the study for PCR O-typing**. Click here for file
