# Supplementary material for: Influence of hydrological conditions on the Escherichia coli population structure in the water of a creek on a rural watershed
Source: BMC Microbiol. 2010 Aug 19;10:222. doi: 10.1186/1471-2180-10-222 (PMC2933670; doi:10.1186/1471-2180-10-222)
Supplement: Additional file 1 — List of primers used in the study for PCR O-typing. [file 1471-2180-10-222-S1.DOC]

**Additional file 1.**

**Primers used in the study for PCR O-typing.**

| Primer designation | Primer sequence | Target | Size of PCR product (bp) | Reference |
| --- | --- | --- | --- | --- |
| gndbis.f | 5’-ATACCGACGACGCCGATCTG-3’ |  |  | Clermont *et al.,* 2007 |
| rfbO7.r | 5’-CGAAGATCATCCACGATCCG-3’ | *rfbO7* | 722 | Clermont *et al.,* 2007 |
| rfbO8a.r | 5’-GAACAATATTGTAAGGTCGCC-3’ | *rfbO8* | 227 | This study |
| rfbO15.r | 5’-GTTTACGTTCCCACCTTATG-3’ | *rfbO15* | 486 | Clermont *et al.,* 2007 |
| rfbO26.r | 5’-GTATGAGCAAAATGGTGAGC-3’ | *rfbO26* | 329 | This study |
| rfbO40.r | 5’- CAGGAAAGCCTCACTATTGG-3’ | *rfbO40* | 625 | This study |
| rfbO45b.r | 5’-TGCGAGTAGACTATCTCAAG-3’ | *rfbO45* | 436 | This study |
| rfbO78.r | 5’-GCACTGCCATTGGTATTTACG-3’ | *rfbO78* | 464 | This study |
| rfbO81.r | 5’-GAGCAGTATATATTACTGGTG-3’ | *rfbO81* | 383 | Clermont *et al.,* 2008 |
| rfbO88.r | 5’-AAGGAAAAACGCTGGGAGAG-3’ | *rfbO88* | 494 | This study |
| rfbO103.r | 5’-GAACTTGGATGGAAAGCCTG-3’ | *rfbO103* | 242 | This study |
| rfbO104.r | 5’-TGGCTTAGGATACTTGCAGC-3’ | *rfbO104* | 410 | This study |
| rfbO111.r | 5’-TTGAGTTCTGAGTGGGAAGG-3’ | *rfbO111* | 522 | This study |
| rfbO128.r | 5’-AACTCGGAGAGTTCCCTATG-3’ | *rfbO128* | 319 | This study |
| rfbO150.r | 5’-TAACGCTAGTGGCAGCAATG-3’ | *rfbO150* | 602 | This study |

All *E. coli* B2 strains were tested for the O-type O81. All *E. coli* B1 strains were tested for O7, O8, O15, O26, O40, O45b, O78, O81, O88, O103, O104, O111, O128, O150 O-type by using the molecular O group determination method described by Clermont *et al.* (2007). PCR was performed in three multiplex reactions.
